# Fibroblast Growth Factors and Cellular Communication Network Factors: Intimate Interplay by the Founding Members in Cartilage

**DOI:** 10.3390/ijms23158592

**Published:** 2022-08-02

**Authors:** Satoshi Kubota, Eriko Aoyama, Masaharu Takigawa, Takashi Nishida

**Affiliations:** 1 Department of Biochemistry and Molecular Dentistry, Faculty of Medicine, Dentistry and Pharmaceutical Sciences, Okayama University, Okayama 700-8525, Japan; tnishida@md.okayama-u.ac.jp; 2 Advanced Research Center for Oral and Craniofacial Sciences, Faculty of Medicine, Dentistry and Pharmaceutical Sciences, Okayama University, Okayama 700-8525, Japan; eaoyama@cc.okayama-u.ac.jp (E.A.); takigawa@md.okayama-u.ac.jp (M.T.)

**Keywords:** fibroblast growth factor, cellular communication network factor, cartilage, skeletal development, CCN2

## Abstract

Fibroblast growth factors (FGFs) constitute a large family of signaling molecules that act in an autocrine/paracrine, endocrine, or intracrine manner, whereas the cellular communication network factors (CCN) family is composed of six members that manipulate extracellular signaling networks. FGFs and CCNs are structurally and functionally distinct, except for the common characteristics as matricellular proteins. Both play significant roles in the development of a variety of tissues and organs, including the skeletal system. In vertebrates, most of the skeletal parts are formed and grow through a process designated endochondral ossification, in which chondrocytes play the central role. The growth plate cartilage is the place where endochondral ossification occurs, and articular cartilage is left to support the locomotive function of joints. Several FGFs, including FGF-2, one of the founding members of this family, and all of the CCNs represented by CCN2, which is required for proper skeletal development, can be found therein. Research over a decade has revealed direct binding of CCN2 to FGFs and FGF receptors (FGFRs), which occasionally affect the biological outcome via FGF signaling. Moreover, a recent study uncovered an integrated regulation of FGF and CCN genes by FGF signaling. In this review, after a brief introduction of these two families, molecular and genetic interactions between CCN and FGF family members in cartilage, and their biological effects, are summarized. The molecular interplay represents the mutual involvement of the other in their molecular functions, leading to collaboration between CCN2 and FGFs during skeletal development.

## 1. Interplay by the Members of Two Distinct Families of Proteins in Cartilage

Fibroblast growth factor (FGF) and cellular communication network factor (CCN) families are established groups of proteins with distinct structural and functional properties. As summarized in Table 1, the mammalian FGF family consists of as many as 22 members, whereas the CCN family has six members only. Structurally, FGFs are characterized by a single core region containing conserved amino acid residues for specific binding to FGF receptors (FGFRs) [1]. In contrast, CCNs are generally larger than FGFs in molecular weight and are composed of four conserved modules linked in tandem, all of which bind to a number of cofactors. Therefore, while FGFs are believed to reveal specific effects through their own receptors, CCNs exert diverse effects depending upon the cofactors present in the microenvironment [2]. However, certain members of each family share common functional counterparts that are represented by heparan sulfate proteoglycans and FGFRs, which is introduced later in detail. Moreover, recent studies uncovered that these members from CCN and FGF families directly bind to each other, and one regulates the gene expression of the other. These findings collectively indicate the intimate interplay involved in their molecular function. In cartilaginous tissues, members of both families are present and play crucial roles. Therefore, it is critical to know the FGF–CCN interplay for understanding better their molecular behaviors and biological functions in cartilage.

## 2. FGF Family

The fibroblast growth factor (FGF) family is a large protein family that consists of 18 secretory signaling molecules and four proteins of intracellular functions [3,4]. Although human FGF ligands are numbered from 1 to 23, FGF15 has not been identified, whereas murine genome contains *FGF15* and lacks *FGF18* [1]. Among the 18 extracellular members, most typical members are called canonical FGFs, acting in paracrine and autocrine fashions under the interaction with heparan sulfate proteoglycans in the extracellular matrix (ECM) and various FGF binding proteins. This group includes FGF-1, 2, 4, 8, 9, and 18, which all play critical roles in cartilage development, growth, and metabolism under the interaction with their proper receptors, FGF receptor (FGFR) 1, 2, 3, and 4 [3,4].

The FGFRs are transmembrane molecules with tyrosine kinase activity in the intracellular domains and transmit the signals from high-affinity ligands upon binding to their extracellular domains. Activation of the kinase in the intracellular domain is provoked by the dimerization of the receptors caused by the binding of a proper ligand, leading to the activation of mitogen activated protein kinases (MAPKs), signal transduction, and activation of transcription (STAT) and phospholipase C (PLC) -γ [5,6,7,8,9]. As a result, these 15 canonical FGFs conduct essential cellular events in the early stages of development in a variety of tissues and organs [3,4]. After development, these proteins still act as local regulators of growth, maintenance, and regeneration of corresponding tissues and organs. The other extracellular FGF family members are profoundly involved in phosphate homeostasis, bile acid synthesis, and lipid metabolism as hormones, and are thus entitled endocrine FGFs [10,11,12,13]. Finally, four intracellular members are mainly engaged in the regulation of voltage-gated sodium channels without interaction with the abovementioned FGFRs [5,14,15,16]. Furthermore, it is also known that one of the representative canonical ligands, FGF-2, also executes its intracellular mission in the nucleus [17,18]. Recent studies also uncovered an endocrine function of the other founding member, FGF-1, as a metabolic hormone that prevents the development of insulin resistance [19,20]. In this way, in spite of their structural similarities, the biological functions of FGFs turned out to be quite diverse and beyond the functionality suggested by their original names.

## 3. FGFs in Cartilage

**Figure 1:**
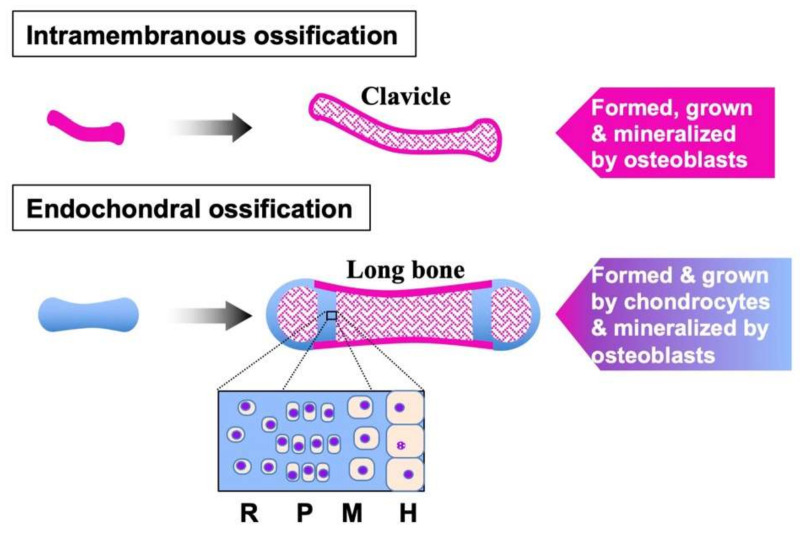
Two major modes of the ossification that constructs the human skeleton. Clavicle and cranial bones are formed through intramembranous ossification, where osteoblasts create the prototype (left) and grow the bone (shown in pink). Most of the other bones are initially formed by chondrocytes as cartilage anlagen (left, shown in light blue), and chondrocytes grow them at the growth plate inside and develop articular cartilage at the epiphysis. Along with bone growth, ECM is mineralized by osteoblasts. In the growth plate, chondrocytes follow a serial differentiation process from resting (R), proliferation (P), mature (M), and hypertrophic (H) stages towards ossification (bottom).

The mammalian skeleton is developed through two distinct procedures; one is intramembranous ossification, and the other is endochondral ossification, in which chondrocytes play a central role (Figure 1) [3,21]. As a typical example, development of appendicular long bones starts from the formation of the limb bud by mesenchymal condensation. Subsequently, all of the appendicular skeletal elements are initially produced as cartilage by the cells grown and differentiated from the limb bud. During this limb bud formation and patterning process, FGF-4 and -8 are known to act as key signaling molecules to determine the shape, size, and location of each skeletal element [4,22]. At this earliest stage of chondrogenesis, FGF-2, -9, and -17 as well as FGF-4 and -8 are produced, suggesting their fundamental role in skeletal development [3]. Endochondral ossification and articular cartilage development start therein from the vascular invasion at the center to construct the primary ossification center.

Thereafter, chondrocytes grow the bone as cartilage, following a series of differentiation steps that construct a distinct structure called growth plate. Immature chondrocytes located distant from the ossification center are at the resting stage and are thus quiescent. Towards the ossification center, those chondrocytes gradually proliferate to increase the number of cells, while forming characteristic columnar structures. After the proliferation stage, chondrocytes maturate and produce cartilaginous ECM. In this way, mature chondrocytes grow the cartilaginous bone anlagen, whereas these cells continue later differentiation to the hypertrophic stage. At this terminal stage of chondrocyte differentiation, enlarged chondrocytes eventually disappear after producing matrix vesicles that initiates calcium crystallization with osteoblasts. Along with the progress of bone growth and ossification, secondary ossification centers are formed at epiphyses, leaving permanent articular cartilage at the surface of the bone to serve as a critical component that supports locomotive function of synovial joints. During this process after early chondrogenesis, expression of FGF-8 with a potential to promote chondrocytic differentiation is quite low, whereas FGF-2 is significantly expressed in these chondrocytes [3,23]. Although FGF-2-null mice show no prominent abnormality in the growth plate, these mice are characterized by decreased bone mass, suggesting the contribution of FGF-2 to proper endochondral ossification [24]. Several studies with isolated chondrocytes have indicated that FGF-2 promoted cell proliferation, whereas this canonical ligand was found to accelerate the degradation of cartilaginous ECM [25,26]. Recent studies also unveiled the catabolic effects of another founding member, FGF-1, on chondrocytes. Of note, FGF-1 is induced in the articular cartilage of a rat model of osteoarthritis [27], in which articular cartilage is damaged, and locomotive function is impaired.

As such, these two founding members of the FGF family are regarded as pathological as well as physiological signaling molecules in cartilage. This notion is consistent with the fact that excess FGF signaling caused by activating mutations in the FGFR3 gene incurs severe defects in endochondral ossification and skeletal development observed in achondroplasia and thanatophoric dysplasia [28,29]. This catabolic outcome of excess FGF signaling is also supported by a report showing the therapeutic potential of an anti-FGFR antibody against osteoarthritis [30]. However, FGFR3 is distinctly demonstrated on chondrocytes in both growth plate and articular cartilage in normal individuals, indicating the physiological role of FGF signaling during cartilage development and maintenance. It is known that FGF-9 and 18 are produced by perichondrium and periosteum, and mice lacking FGF-9 and/or FGF-18 suffer from osteochondral dysplasia [3,31,32]. Additionally, studies in vitro and in vivo suggest the anabolic effects of FGF-18 on chondrocytes [33,34]. These two ligands are thought to regulate endochondral ossification and articular cartilage homeostasis from remote sites in a delicate manner.

## 4. CCN Family

Cellular communication network factor (CCN) is a relatively new family of proteins founded in 1993 simply by combining the initial letters of the first three members [35]. The first member of this family was identified in 1989 [36], and was designated as chicken embryonic fibroblast (CEF) 10 or cysteine-rich protein (CYR) 61. Within a few years, another two proteins with striking structural similarities were discovered and named connective tissue growth factor (CTGF) and nephroblastoma overexpressed (NOV) [37,38]. At this time, these proteins were regarded as members of a distinct protein family, and the family name was given based on the acronym of these founding members (Cyr61-Ctgf-Nov) by Bork in 1993 [2,35]. Unlike the FGF family, this family was not named after CTGF, owing to its molecular property. CCN2 could be regarded as a growth factor, considering that a recombinant CCN2 produced by *E. coli* exerted significant mitogenic effects on osteoblasts [39]. However, such effects observed with CCN2 purified from mammalian cells might involve those of the co-purified minor partners, because it binds to a number of growth factors [37,40]. Furthermore, CCN2 interacts with a variety of cell surface receptors as well as ECM components, indicating its property as a matricellular protein rather than a growth factor [41]. As such, the CCN family was born under a definition that is different from the present one—cellular communication network factors. Together with the additional three members discovered later as Wnt-induced protein 1, 2, and 3, six members finally constitute the mammalian CCN family [42]. Subsequently, Brigstock comprehensively explained the concept of the CCN family in 1999 [43], and two years later, this nomenclature was adopted in 2001 by the International CCN Society [44].

**Figure 2:**
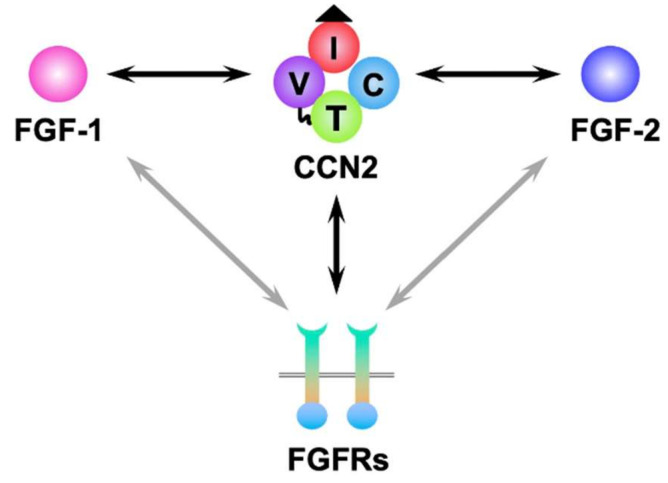
Molecular structure of CCN2 and its interactions with canonical FGFs and their receptors. Tetra-modular structure of CCN2 with a signal peptide for secretion (black triangle) and a hinge domain (wavy line between V and T) is illustrated at the middle of the top. I, V, T, and C represent insulin-like growth factor binding protein-like (IGFBP), von Willebrand factor type C repeat (VWC), thrombospondin 1 type 1 repeat (TSP1), and C-terminal cystine knot (CT) modules, respectively. Bi-directional arrows denote direct binding between the two. Out of four FGF receptors, FGFR1, -2 and -3 are all known to bind to CCN2.

Although functional identification of a signal peptide was reported only in CCN2 [45], all of the CCN family members contain deduced signal peptides for secretion at the N-termini and thus basically are extracellular entities. Nevertheless, a few members are suggested to also work inside the cells, particularly in the nucleus. Indeed, CCN5 was shown to be recruited to the promoter of the transforming growth factor-β receptor II gene and to repress its expression in human breast cancer cells [46]. Nuclear localization of CCN2 and CCN3 was also confirmed in human mesangial cells and osteosarcoma cells, respectively [47,48]. Following the signal peptide, three or four cysteine-rich modules that are highly conserved among the members are connected in tandem. These modules are designated based on the structural characteristics: N-terminus, insulin-like growth factor binding protein-like (IGFBP), von Willebrand factor type C repeat (VWC), thrombospondin 1 type 1 repeat (TSP1), and C-terminal cystine knot (CT) (Figure 2) [2,40,49,50]. Except for the hinge domain between the VWC and TSP1 modules, no other domains are present. Using these highly interactive modules, CCN family proteins bind to a variety of molecules in the extracellular microenvironment, such as growth factors, cell surface receptors, and ECM components [40,41]. As a result, these proteins manipulate and tune the extracellular signaling network, yielding highly context-dependent biological effects. Considering these unique molecular behaviors, in 2018 the CCN family was reborn as the cellular communication network factor family under the approval of the human genome organization (HUGO) upon proposal by the international CCN society (ICCNS) scientific committee [51]. Collectively, the naming of “CCN” was proposed under a group effort by a number of scientists.

## 5. CCNs in Cartilage

During the development of long bones through endochondral ossification, all of the CCN family members are differentially expressed, suggesting certain roles of all the members in this process [52]. In developing cartilage anlages, CCN1 is detected broadly in the proliferating and pre-hypertrophic zones, whereas CCN2 is present in the pre-hypertrophic and hypertrophic zones around the vascular invasion sites. CCN3 is initially induced in the regions deep inside, where ossification starts. Thereafter, this protein stays at the end of the initial hypertrophic layer [53]; however, accumulation of CCN3 is mainly observed in the zones with immature chondrocytes. Both CCN4 and CCN5 are observed in the pre-hypertrophic and hypertrophic zones, and CCN6 is found around the vascular invasion sites [52].

In articular cartilage left after the completion of skeletal development, CCN1 and CCN2 are present, even under normal conditions, and are increased by the development of osteoarthritis [53,54]. This induction is considered to be a reparative response against the cartilage damage toward regeneration. In fact, exogenous addition of CCN2 or its modular fragments onto the damaged articular cartilage accelerated its regeneration [55,56]. All of the other members are not actively produced; however, local accumulation of CCN3 in the restricted area between the surface and middle zones is observed [21].

Consistent with their distribution, *Ccn2*-null mice are lethal upon delivery due to the severe defect in endochondral ossification [52], and mice with CCN3 with the deletion of the VWC module develop severe joint malformation [57]. It should also be noted that cartilage-specific overexpression of CCN2 conferred resistance to osteoarthritis in mice [58]. In contrast, no distinct phenotypic changes were evident in the cartilage of mice lacking CCN4, CCN5, or CCN6 [59,60,61], except for the expanded hypertrophic zone in the CCN4-deficient mice [59]. Owing to the indispensable role of CCN1 in vascular development, *Ccn1*-null mice are embryonic lethal, and thus sufficient information on the effect of CCN1 depletion in cartilage development was not obtained. Nevertheless, a subsequent study revealed that cartilage-specific overexpression of CCN1 resulted in chondrodysplasia, indicating its significant role in cartilage development and maintenance [54].

CCN1 and CCN2 were reported to promote the proliferation and maturation of chondrocytes [54,62], while CCN3 represses their proliferation and regulates their commitment to articular chondrocytes [52,63] On the other hand, these three founding members uniformly promote angiogenesis [64,65,66]. Collectively, CCNs collaborate to initiate endochondral ossification through the formation of ossification centers by their angiogenic activity. Afterwards, CCN3 is used to keep chondrocyte resting at an early stage, while CCN1 and CCN2 are produced at later stages to promote endochondral ossification [54,67]. In adult articular cartilage, CCN3 distributed beneath the surface layer is thought to maintain the quiescence and stemness of the surrounding articular chondrocytes [21], because CCN3 is known to be a critical regulator of cellular stemness [68,69]. CCN1 and CCN2 are found therein, probably to support the potential to proliferate and supply the cartilaginous ECM, which is, however, insufficient to combat osteoarthritis per se.

## 6. Molecular Interaction between FGFs and CCNs

Because interaction with heparan sulfate proteoglycans is one of the molecular events involved in the function of matricellular CCNs and FGFs [3,41], indirect association between CCNs and FGFs is supposed in various physiological and pathological situations in vivo. In fact, CCN1 was reported to displace FGF-2 from the ECM of human umbilical vein endothelial cells, leading to the enhancement of FGF-2 function [70]. It is also known that both CCN2 and FGF-2 are rapidly secreted from injured cartilage, indicating their common matricrine action [71,72]. Furthermore, experiments in vitro showed direct interaction among CCNs, canonical FGF ligands, and receptors. Direct binding between CCN1 and FGF-2 was first indicated by a solid-phase binding assay, which was not affected by the enzymatic fragmentation of CCN1 by kallikrein-related peptidase 12 [73]. The direct interaction of CCN2 with FGF-2 was more carefully confirmed by surface plasmon resonance (SPR) and co-immunoprecipitation analyses, as well as a solid-phase binding assay with a distinct system [74]. As a result, a dissociation constant (Kd) of 5.5 nM was computed for the direct interaction between CCN2 and FGF-2 [74]. Extensive evaluation with mono-modular, di-modular, and tri-modular fragments of CCN2 clarified that the CT module was the binding interface between the two [74]. Under a similar experimental strategy, binding between FGF-1 and CCN2 was subsequently confirmed [75]. Namely, FGF-1 was found as a candidate of the CCN2 binding counterparts by screening a protein array, which was subsequently confirmed by a solid-phase binding assay. The result of SPR analysis revealed a Kd of 3.98 nM for FGF-1-CCN2 interaction [75]. Therefore, FGF-1 and FGF-2 share CCN2 as a common functional counterpart, although it is not clear whether these FGFs compete each other to obtain CCN2 as a partner, or not. Additionally, binding between CCN5 and FGF-2 was also indicated and was shown to be sensitive to the proteolytic cleavage of CCN5 at the TSP1 module [73].

It is a matter of fact that FGF-1 and FGF-2 bind to their canonical receptors. Moreover, CCN2 was found to bind to FGFR1, FGFR2, and FGFR3 [74,76], whereas, to our knowledge, interaction between CCN2 and FGFR4 has not been reported. Proteomic screening of CCN2-binding molecules nominated FGFR2 and FGFR3 as two of the top-three candidates for CCN2 partners among the FGF-related proteins tested [76]. These interactions were confirmed by solid-phase binding assays, which indicated a stronger affinity of FGFR2 to CCN2 than FGFR3. According to the result of SPR analysis, the Kd between CCN2 and FGFR2 was 7.91 nM. Interestingly, CCN2 rather enhanced the molecular association between FGFR2 and its canonical ligands, FGF-2 and FGF-4, suggesting their collaboration [76]. Actually, canonical FGF signaling as represented by MAPK activation was enhanced by the addition of CCN2 to osteoblastic cell culture [76]. FGFR1 was not included as one of the highly-ranked candidates in the proteomic screening above; however, direct interaction of this FGF receptor with CCN2 was solidly confirmed by the combination of solid phase, SPR, and co-immunoprecipitation analyses [74]. These direct interactions are summarized in Figure 2, with CCN2 at the center.

## 7. FGF–CCN Collaboration in Cartilage

In the growth plate cartilage, resting chondrocytes express FGFR2 at a modest level, whereas proliferating chondrocytes demonstrate abundant FGFR3 [3]. Afterwards, expression of this receptor decreases before hypertrophy, and pre-hypertrophic and hypertrophic chondrocytes show high levels of FGFR1 demonstration [3]. Because FGF-1, CCN2, and these FGFRs are distributed in the growth plate, the interactions of these signaling molecules and receptors are involved in the process of endochondral ossification in a zone-specific manner. FGFR3 displayed on proliferating chondrocytes transmit signals from FGF-2 to promote the proliferation of immature chondrocytes in this zone through FGFR3, where CCN2 expression is not prominent, and thus the biological significance of CCN2–FGF-2 molecular interaction is obscure. However, as stated in the last section, CCN2 expression is dramatically induced before hypertrophic differentiation, while FGFR1 is dominantly demonstrated on the chondrocytes in the pre-hypertrophic and hypertrophic zones. Therefore, the physiological significance of CCN2–FGF-2–FGFR1 interaction is strongly suggested therein.

**Figure 3:**
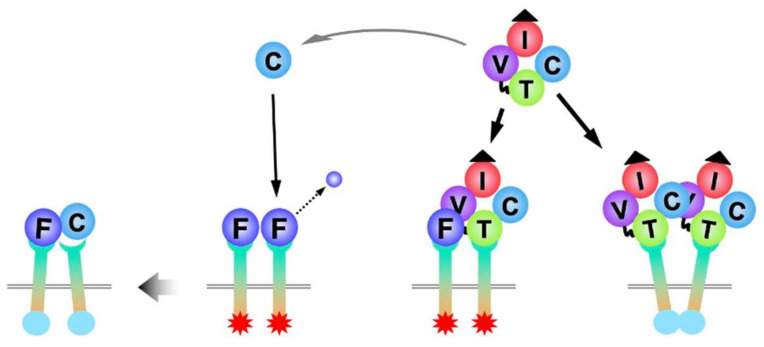
Multimolecular interactions among FGF-2, FGFR1, CCN2, and its single modular sub fragment. Activated form of FGFR1 is represented by stars in red, whereas silent form is represented by spheres in light blue. Letters indicate the same modules defined in Figure 2, with the exception of F, which denotes FGF-2.

Considering the restricted expression of FGFR1 in hypertrophic chondrocytes, the FGF signal through this particular receptor is supposed to play a central role in the terminal differentiation of chondrocytes in this zone. Regarding the ligands, both FGF-2 and CCN2 bind to FGFR1 and promote the hypertrophic differentiation of chondrocytes, enhancing the expression of hypertrophic marker genes. Interestingly, although CCN2 binds to FGFR1, this molecule neither provokes FGF signaling by itself, nor acts as an antagonist that interferes with the activation of FGFR activation by FGF-2, suggesting that FGF-2–CCN2 heterodimer is also capable of activating FGFR1 (Figure 3) [74]. In addition, a recent study revealed that CCN2 can be proteolytically processed into biologically active C-terminal sub-fragments that comprise TSP1 and CT modules [77]. Generation of such C-terminal fragment has actually been confirmed in human chondrocytic cell cultures [74,78]. Moreover, a CCN2 fragment containing the CT module was found to directly bind to FGF-2, whereas the TSP1 module did not [74]. Importantly, this CT module does not bind to FGFR1, but inhibited the signal transduction from FGF-2 to the downstream MAPKs [74]. Excess and early canonical FGF signal during endochondral ossification results in the compression of the growth plate, as observed in achondroplasia. CCN2 is produced from the chondrocytes before hypertrophic differentiation and in regulating the proper onset of this terminal cellular event, probably via the formation of C-terminal sub-fragments that regulate the signal through FGFR1.

Under physiological conditions, FGF-1 and CCN2 do not usually meet in cartilage. Nevertheless, FGF-1 was reported to appear in articular cartilage upon cartilage injury and provoke catabolic response and ectopic hypertrophy in articular chondrocytes, leading to osteoarthritic cartilage degeneration [27]. Of note, FGFR1 is demonstrated on articular chondrocytes, and the signals through this receptor are indicated to promote cartilage degradation. Exogenous application of CCN2 is known to ameliorate this pathological state of articular cartilage in vivo [55], where CCN2, FGF-1, and FGFR1 could meet, and their direct interaction is assumed, but its biological significance is still unclear. In articular cartilage, the co-presence of CCN2 and FGF-2 was indicated in the pericellular matrix of chondrocytes [72], and the roles of these two proteins in osteoarthritis development is highly controversial. In one study, postnatal and cartilage-specific deletion of floxed CCN2 by tamoxifen-inducible *Cre* under the control of an *Acan* promoter conferred no significant effect on OA development [79], and global CCN2 deletion after birth by human ubiquitin C promoter-driven *Cre* even added resistance against OA in the other [72]. The difference between the two suggests some role of CCN2 acting in paracrine and/or endocrine fashions in OA development. However, paradoxically, constitutive overexpression of CCN2 by a *Col2a1* promoter protected the cartilage from OA [54]. Of interest, deletion of the larger isoform of FGF-2 protected mice from OA, whereas ablation of the smaller isoform accelerated OA development [80]. These findings may represent isoform- and context-dependent roles of FGF-2 as well as CCN2, behind which the intimate interplay among these ligands and receptors exists.

## 8. FGF–CCN Genetic Interaction in Chondrocytes

**Figure 4:**
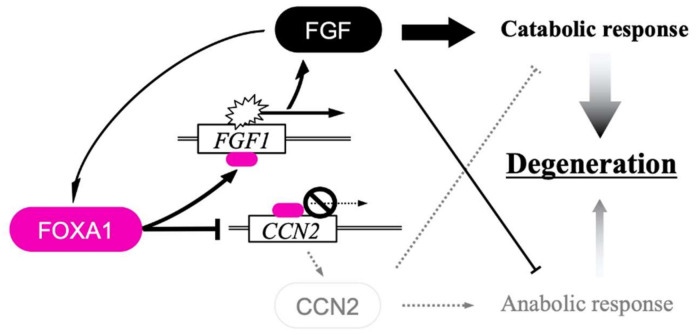
Integrated bidirectional regulation of *FGF1* and *CCN2* by the FGF signal in chondrocytes. Canonical FGF signaling emitted by FGF-1 induces FOXA1 to activate *FGF1* and repress *CCN2* simultaneously. Activation of *FGF1* results in the formation of a positive feedback loop to amplify the outcome of this bipartite regulation. Synergistic cartilage degeneration would be induced by enhancing the catabolic effects of the FGF signal and repressing the anabolic effects of CCN2. Objects on *FGF1* and *CCN2* stand for transcriptional co-activator and co-repressor, respectively.

To date, no reports directly show regulation of the gene expression of FGF family members by a CCN family member, despite evidence that indicates possible intracrine function of a few CCN family members as transcription factors [46,47,48]. Similarly, little information has been available on the regulation of CCN family gene expression by the FGF signaling. Nevertheless, recent studies revealed a clear-cut regulation of *CCN2* at a transcriptional level by canonical FGF signaling in chondrocytes [81]. Namely, FGF-1, one of the founding members of the FGF family, was found to strongly repress the expression of this CCN family founder. This effect of FGF-1 was accompanied by other catabolic responses represented by the strong induction of MMP-13 expression and decreased expression of the genes encoding cartilaginous ECM components. At the same time, FGF-1 expression was strongly enhanced, which amplified the outcome of the FGF signaling through a positive feedback loop (Figure 4). Surprisingly, this simultaneous *CCN2* repression and *FGF1* induction by FGF-1 were mediated by the same transcription factor, forkhead box protein A1 (FOXA1) [81]. FOXA1 is a representative member of a huge family of transcription factors. The FOX family is divided into 19 subgroups that start from FOXA group containing this protein as the first member [82]. Using their forkhead DNA binding motifs, these proteins bind directly to DNA in the chromatin structure, and FOXA1 indeed binds to the proximal enhancers in both *CCN2* and *FGF1* loci [82]. Regarding this bidirectional effect mediated by a single transcription factor, it is of particular note that FOXA1 either enhances or represses the target gene expression, depending upon the biological context and transcriptional microenvironment. Physiologically, this bipartite regulation could be a critical component of the biological system that induces hypertrophy in growth plate chondrocytes. Upon the hypertrophic differentiation of chondrocytes, FGFR1 emerges and MMP-13 is induced with a rapid decrease in *CCN2* expression after reaching the peak at the pre-hypertrophic stage. From a pathological point of view, this bidirectional regulation by FOXA1 is highly suspected to be involved in the development of osteoarthritis. FGF-1 induces catabolic responses in chondrocytes, which is amplified by the positive feedback loop, together with the simultaneous suppression of a cartilage regenerative factor, CCN2 (Figure 4). This event can be triggered by another FGF family member, FGF-8, which was induced along with the osteoarthritis development in a mouse model [83,84].

It is not clear whether this FOXA1-mediated FGF–CCN2 genetic interaction is working specifically in chondrocytes or is a universal molecular events in various types of cells. It is known that FOXA1 binds to *CCN2* and *FGF1* in HEPG2 hepatocellular carcinoma cells. Metabolically, CCN2 supports glycolysis in chondrocytes, whereas FGF-1 suppresses lipolysis and hepatic glucose production. Therefore, this FGF–CCN2 genetic interaction may be utilized in a number of cells, particularly in hepatocytes, in order to maintain the systemic homeostasis of energy metabolism.

## 9. Mechanism of the Integrated FGF–CCN Regulation in Chondrocytes

The mechanism of how a single transcription factor, FOXA1, could regulate two distinct genes, *FGF1* and *CCN2*, is not yet clear. It is at least indicated that class I histone deacetylases (HDACs) plays a significant role, both in *FGF1* upregulation and *CCN2* down regulation, because the bipartite effects of FGF-1 were abolished by class I HDAC inhibitors [81]. It is probable that HDACs contributes to the formation of the general chromatin status required for transcriptional regulation in concert with FOXA1. This finding is consistent with the molecular property of FOXA1 as a pioneer factor that opens the condensed chromatin into an accessible form [85]. Thereafter, in order to realize this bidirectional gene regulation distinctively, efficient and precise recruitment of target-specific cofactors is required. Although a number of transcription factors were reported to interact with and regulate *CCN2* and *FGF1*, it is unclear which factors are recruited to each locus due to the activation of canonical FGF signaling. Nevertheless, it is suspected that FOXA1 plays a crucial role other than as a pioneer factor in the stable locus-dependent recruitment of cofactors.

Highly locus-specific transcriptional regulation is thought to be enabled within a membrane-free microcompartment known as liquid droplets, which are constructed through the liquid–liquid phase separation (LLPS) process [86]. Most of the membrane-free subcellular structures in the nucleus and cytoplasm, such as nucleoli, paraspeckles, Cajal bodies, nuclear speckles, and stress granules, are formed through this physicochemical process. LLPS is also utilized for realizing independent transcription regulations occurring simultaneously in the nucleus [87]. Into the droplet formed around a locus, the factors necessary for the proper transcriptional regulation are recruited and condensed without being mixed up with other proteins in the nucleus. To undergo LLPS, two critical components are required; one is proteins with intrinsically disordered regions (IDRs), and the other is RNA molecules represented by long noncoding RNAs (lncRNAs) [88]. In this context, it is of particular note that RNA polymerase II, which produces mRNAs and lncRNAs, was shown to induce LLPS via its IDR [89], suggesting that LLPS may take place around all the transcriptionally active loci of protein coding genes. Furthermore, we found that FOXA1 could be an IDR protein by analyzing the primary structure of this protein in silico (unpublished data). Owing to the presence of FOXA1, droplets around *CCN2* and *FGF1* could be more stable, providing a closed nano environment, in which each gene can be regulated in a highly independent fashion. As such, not only as a pioneer factor to open the chromatin up, FOXA1 may now be regarded as a nuclear mediator that contributes to the clear-cut bidirectional regulation of the target genes by promoting LLPS, supporting the genetic interaction of *CCN2* and *FGF1* with its autoinduction loop. Considering these functionalities of FOXA1, this protein does not seem to be involved in the selective recruitment of cofactors for *CCN2* and *FGF1* loci. It is probable that RNA, the other principal component of droplets, is playing a certain role in the locus and context-dependent selection of cofactors to be invited therein.

## 10. Conclusions

The molecular interaction of CCN2 with FGF family members other than FGF-1 and FGF-2 has not been investigated. However, as CCN2 binds directly to three out of four FGFRs and HSPG, the involvement of CCN2 in the events caused by all of the canonical and hormone-like FGF ligands is expected. Especially, interaction between CCN2 and Klotho co-receptors appears to be worth investigating, because it could enable tissue-specific response to endocrine FGF-1, FGF-19, FGF-21, and FGF-23 through the regulation of CCN2 production. From a metabolic point of view, direct and indirect interplay between CCN2 and FGF-21, as well as FGF-1, is an interesting topic. In this context, CCN3, which is a CCN family member with molecular functionalities counteracting CCN2, needs to be investigated, as the hormonal function of CCN3 and its association with obesity, diabetes, and insulin resistance were pointed out.

In addition to FGF-1, FGF-2, FGFR1, FGFR2, and FGFR3, proteomic analysis picked up FGF-10, FGF-12, FGF-13, and FGF-16 as potential candidates that may bind to CCN2 [70]. In spite of there being no evidence indicating the molecular interactions between FGFs/FGFRs and CCN family members except for CCN2, direct binding of the other CCN family members with FGF ligands and FGFRs is highly suspected, considering the molecular nature of CCN family members with four highly interactive modules. Regarding indirect binding, CCN1 and CCN3 are able to interact with FGF ligands and receptors via HSPG. However, only a few reports, including a very recent one indicating the regulation of CCN2 by FGF-18, have unveiled the genetic interaction between FGFs and CCN2 only. Proteomic and other genome-wide analyses are necessary in order to obtain an integrated view of the molecular and genetic interplay between these two families of signaling molecules.

## Figures and Tables

**Table 1:** Distinct properties of mammalian CCN and FGF family members.

Properties	FGF Family	CCN Family
Family members	22	6
Molecular weights	17–34 kD	^1^ 26–42 kD
Molecular structure	Single core region	Four modules linked tandem
Receptors	Specific	Multiple and diverse
Function	Specific	Context-dependent

^1^ Molecular weights of the major isoforms.
